# Comparative Effects of Deoxynivalenol, Zearalenone and Its Modified Forms De-Epoxy-Deoxynivalenol and Hydrolyzed Zearalenone on Boar Semen In Vitro

**DOI:** 10.3390/toxins14070497

**Published:** 2022-07-18

**Authors:** Panagiotis D. Tassis, Nicole Reisinger, Veronika Nagl, Eleni Tzika, Dian Schatzmayr, Nikolaos Mittas, Athina Basioura, Ilias Michos, Ioannis A. Tsakmakidis

**Affiliations:** 1 Farm Animals Clinic, School of Veterinary Medicine, Aristotle University of Thessaloniki, 54627 Thessaloniki, Greece; eltzika@vet.auth.gr (E.T.); imichos84@hotmail.com (I.M.); iat@vet.auth.gr (I.A.T.); 2 DSM-BIOMIN Research Center, Technopark 1, 3430 Tulln, Austria; nicole.reisinger@dsm.com (N.R.); veronika.nagl@dsm.com (V.N.); dian.schatzmayr@dsm.com (D.S.); 3 Department of Chemistry, School of Science, International Hellenic University, 65404 Kavala, Greece; nmittas@chem.ihu.gr; 4 Department of Agriculture, School of Agricultural Sciences, University of Western Macedonia, 53100 Florina, Greece; abasioura@uowm.gr

**Keywords:** deoxynivalenol, de-epoxy-deoxynivalenol, zearalenone, hydrolyzed zearalenone, boar, semen, spermatozoa, mycotoxins, reproduction, swine

## Abstract

Deoxynivalenol (DON) and zearalenone (ZEN) are described as detrimental factors to sow and boar fertility. In comparison, literature reports on the impact of modified forms of DON and ZEN, such as de-epoxy-DON (DOM-1) and hydrolyzed ZEN (HZEN), on swine reproduction are scarce. The aim of our study was to compare the effects of DON, DOM-1, ZEN and HZEN on boar semen in vitro. To this end, pooled boar semen ejaculates from two adult boars were treated with either 50.6 μM DON, 62.8 μM ZEN or equimolar concentrations of DOM-1 and HZEN, respectively (dilution volume of *v*/*v* 0.7% DMSO in all cases). Effects on semen motility, morphology, viability, hypo-osmotic swelling test reaction and DNA integrity were investigated hourly up to four hours of incubation. DON negatively affected particular parameters evaluated with a computer-assisted sperm analysis system (CASA), such as immotile spermatozoa and progressive motile spermatozoa, whereas those effects were absent in the case of DOM-1 treatment. In contrast to HZEN, ZEN affected almost all CASA parameters. Furthermore, only ZEN decreased the proportion of viable spermatozoa and increased the proportion of spermatozoa with abnormalities. In conclusion, DON and ZEN negatively affected boar semen in vitro, whereas equimolar concentrations of DOM-1 and HZEN did not induce harmful effects.

## 1. Introduction

The *Fusarium* mycotoxins deoxynivalenol (DON) and zearalenone (ZEN) have been heavily investigated in the past half-century with regard to their possible detrimental effects on swine health and reproduction. They are both frequent contaminants of grains worldwide and often coexist in contaminated commodities [1].

DON belongs to the trichothecene family of mycotoxins and has been proven to affect the reproductive performance of pigs. In particular, effects of DON include the impairment of oocyte maturation and embryo development (induction of abnormalities of the meiotic spindles and alteration of oocyte cytoplasmic maturation) [2,3,4], induction of autophagy/apoptosis and epigenetic modifications in porcine oocytes [5], or alteration of porcine granulosa cell proliferation (biphasic effect: lower concentration (0.034 mM) of DON results in increased proliferation, but greater concentration (3.4 mM) has the opposite effect) [6]. Moreover, DON affected boar semen characteristics in vitro either alone (50.6 μΜ) or after simultaneous exposure to ZEN (50.6 μM DON and 62.8 μM ZEN) [7].

After ingestion, DON can be metabolized by intestinal microbes to de-epoxy-DON (DOM-1) that can be found in plasma or excreta [8]. In pigs, microbial formation of DOM-1 is discussed to be acquired and/or age-related, and mainly occurs in the distal part of the digestive system, whereas DON is predominantly absorbed in the upper digestive tract, thus partially “avoiding” microbial de-epoxidation [8,9]. DOM-1 lacks the 12,13-epoxide group, which is regarded as essential for toxicity [10]. As shown in multiple cell lines, toxic effects of DOM-1 are either absent or minimal in comparison to its parent toxin [11,12,13,14]. For example, Dänicke et al. [12] reported that DOM-1 up to concentrations of almost 23 μM did not affect the viability of peripheral blood mononuclear cells (PBMCs) and pig intestinal cells (IPEC-1, IPEC-J2), and Pierron et al. [13] described that 10 μM DOM-1 did not upregulate mRNA relative expression levels of proinflammatory cytokines and chemokines in pig jejunal explants, in contrast to the effect of equimolar DON concentration.

On the other hand, concerns about the biological activity and toxic potency of DOM-1 on bovine reproduction were raised by Guerrero-Netro et al. recently [15,16]. It was shown that DOM-1 can induce cessation of follicular growth and reduction of bull spermatozoa motility in vitro. Unfortunately, effects of DON on the above-mentioned parameters were not presented. On the contrary, in an in vivo study on pigs, DOM-1 seemed to induce minimal intestinal or liver toxicity, whilst in comparison, DON caused significantly greater toxic effects. However, DOM-1, similarly to DON, increased histological lesions and cell proliferation in lymph nodes [17]. According to a study with PBMCs, DOM-1 did not affect bovine PBMCs but reduced the proliferation of chicken and porcine PBMCs at the highest tested concentration (357 μM), whilst DON heavily affected all species PBMCs at markedly lower concentrations (significant reduction of porcine PBMC proliferation at 0.84, 1.69 and 3.37 μM DON), showing greater inhibitory effects on bovine cells [18].

ZEN predominantly affects the reproductive system of swine by binding to estrogen receptors (ERs), with a stronger affinity to ER-α compared to ER-β [19]. In mammals, important metabolites of ZEN are α-zearalenol (α-ZEL) and β-zearalenol (β-ZEL), whilst metabolites of minor importance are α-zearalanol, β-zearalanol and zearalanone (phase I metabolism). Conjugates of ZEN or its major metabolites include glucosides of plant origin, sulfates and glucosides of fungal origin, and glucuronides and sulfates (phase II metabolism) of mammalian origin [19,20,21,22]. Pigs are particularly sensitive to ZEN, mainly because ZEN is predominantly metabolized to α-ZEL in that species, which shows greater estrogenic potency than its parent toxin [20,23,24]. The ability of ZEN to reduce fertilization and normal embryonic development, resulting in significant reproductive disorders, has been proven through a large number of in vitro and in vivo studies in swine [19,25,26]. Furthermore, the negative impact of ZEN on boar semen characteristics and fertilizing ability has been demonstrated in vivo, by reduced serum testosterone levels and libido, decreased testis weights and spermatogenesis [27,28,29], as well as in vitro, with reduced motility, viability and ability of spermatozoa to bind to the zona pellucida, or effects on sperm chromatin integrity [7,30,31,32,33].

The estrogenic potency of hydrolyzed ZEN (HZEN) was evaluated in the study by Fruhauf et al. [34]. HZEN exhibited markedly reduced estrogenicity in vitro and in vivo (50–1000 times less estrogenic potency) when compared with ZEN. After in vivo evaluation in female piglets, HZEN did not affect reproductive tract morphology or expression of ZEN-responsive RNA transcripts in pigs [34].

Taken together, previous research shows that DOM-1 and HZEN exhibit significantly reduced biological activity compared to their parent toxins. In the case of DOM-1, certain studies suggest that this modified mycotoxin retains a part of DON’s toxic potency. So far, neither DOM-1 nor HZEN has been examined for their impact on boar semen characteristics. The present study aims to determine the effects of DON, DOM-1, HZEN and ZEN on boar semen in vitro, thus broadening our understanding of the toxicity of the modified mycotoxins.

## 2. Results

In the experimental set-up, a control group that received neither solvent nor mycotoxins was included. Due to the absence of any significant difference (comparisons among all time points and parameters) between the aforementioned control group and the solvent group (0.7% DMSO), the following sections demonstrate evaluations of the DMSO group and the mycotoxin-treated groups only.

### 2.1. Effects of DON and DOM-1 on Boar Semen Characteristics

#### 2.1.1. Computer-Assisted Sperm Analysis System (CASA) Results

The effects of DON (50.6 μΜ) or equimolar DOM-1 concentrations at five time points of observation (0–4 h) on boar semen motility parameters, assessed with the CASA system, are presented in Table 1 and Table 2. An increase in immotile spermatozoa was observed after DON exposure when compared with the DMSO group (*p* < 0.05). An effect of time irrespective of the treatment in all groups was observed only between the 1st and 2nd hours of the experiment. Quite similarly to the immotile spermatozoa parameter, a significant reduction due to DON exposure is reported for progressive motile spermatozoa, when compared with the DMSO group. An effect of time, as mentioned above, was present on this parameter until the 1st hour of the experiments. On the contrary, DOM-1 did not induce significant negative effects on the above-mentioned parameters. Furthermore, significant effects of DON or DOM-1 were absent in the rest of the investigated CASA parameters.

#### 2.1.2. Results on Morphology, Viability, Hypoosmotic Swelling Test (HOST) and Nuclear Chromatin Integrity

Sperm morphology and viability alterations, as well as HOST results, after DON or DOM-1 (50.6 µM, respectively) exposure of boar semen at five time points of observation (0–4 h) are presented in Table 3. Results of sperm quality characteristics demonstrated the absence of significant negative effects of DON or DOM-1 on viable spermatozoa, head abnormalities and HOST evaluations. Almost all results of all groups, time points and replicates suggested the absence of DNA damage either at the 0 or 4th hour of the study. Only 1 case in the DON group out of a total of 100 evaluations had an almost negligible shift from normality, with 0.5% damage.

### 2.2. Effects of ZEN and HZEN on Boar Semen Characteristics

#### 2.2.1. CASA Results

The effects of ZEN or HZEN (62.8 μΜ, respectively) exposure on boar semen at five time points of observation (0–4 h) with regard to motility characteristics assessed by the CASA system, are presented in Table 4 and Table 5 Results showed a negative effect after ZEN exposure on all but one (amplitude of lateral head displacement (ALH)) parameter. Immotile spermatozoa, straight-line velocity (VSL), linearity (LIN) and wobble (WOB) values were either reduced (VSL, LIN) or increased (immotile, WOB) from 1 h until the end of observations (4 h). On the contrary, HZEN exposure did not affect any motility parameter, when compared with the DMSO group.

#### 2.2.2. Results on Morphology, Viability, HOST and Nuclear Integrity

Boar semen morphology, viability and HOST evaluations, after ZEN or HZEN (62.8 µM, respectively) exposure at five time points of observation (0–4 h) are presented in Table 6. A significant reduction of spermatozoa with normal morphology was present in the ZEN-treated group when compared with the DMSO group. A time effect on the specific parameter was observed up to the 1st hour of the experiments, irrespective of treatment.

Greater proportions of spermatozoa with head abnormalities and a significant reduction of viable spermatozoa are reported for the ZEN group in comparison with the DMSO group. In contrast, HZEN did not induce any morphological or viability alterations when compared with the DMSO group. Mean numbers of HOST-reacting spermatozoa were not affected by ZEN or HZEN. With regard to sperm nuclear chromatin integrity, only three ZEN group replicates had an almost negligible shift from normality. One percent damage was detected at 0 h in two cases and at the 4th hour in another case.

## 3. Discussion

The present study compared the effects of DON, ZEN and their modified forms DOM-1 and HZEN on boar semen in vitro. Results confirm that DON and ZEN elicit toxic effects on boar semen, whilst significant effects were absent for the respective modified mycotoxins. ZEN showed stronger toxic potency than DON in a greater number of parameters of boar semen. Our results agree with previous studies demonstrating ZEN as a major factor that deteriorates boar sperm motility and viability characteristics [7,30,31,32,33].

Comparison of the effects of DON and DOM-1 showed that only DON induced a toxic effect on boar semen, as demonstrated by the alterations of two critical motility parameters of boar semen (i.e., immotile and progressive motile spermatozoa). The DON concentration level used (minimum dose (MiD)) proved to be an approximate entry-level concentration, capable of inducing a toxic effect, since the rest of the CASA parameters tested remained unaffected. Alterations of sperm membrane or chromatin integrity can be associated with such effects on motility parameters. However, in the present study, such etiology for the observed alterations in immotile and progressive motile spermatozoa was not supported by HOST or acridine orange test (AOT) results. The absence of significant effects on membrane or nuclear chromatin integrity can be related to the fact that boar sperm protamine molecules contain 10 cysteine groups each (higher than other species), the chromatin crosslinking in boar sperm is greater than in other mammalian species and DNA shows greater stability [35]. On the other hand, a different possible etiology of motility alterations could include a disturbance of cell physiological status due to impaired sperm mitochondria function or sperm membranes’ lipid peroxidation, variables that were not evaluated in this study. Such a hypothesis should be further evaluated in future studies.

Our study is the first that investigated DOM-1 and HZEN effects on boar semen in vitro. A DOM-1 concentration equimolar to DON failed to induce any toxic effect on boar semen characteristics. Considering that the exposure level of DON used in this study corresponds to extremely high levels of DON in feed [36], it can be deduced that DOM-1 will not affect boar semen under realistic field conditions.

The effects of DON and DOM-1 were previously assessed in various cell lines from different tissues and species (trout gill (RTgill-W1), pig intestinal cells (IPEC-1 and IPEC-J2), mouse macrophages RAW 264.7, human liver cells (HepG2)) [37]. DON reduced viability in RTgill-W1 (10 μM), IPEC-1 (above 0.9 μM), IPEC-J2 (above 3.5 μM) and HepG2 cells (above 0.9 μM), whereas DOM-1 did not have such an effect up to 228 μΜ. Similarly, albumin secretion of HepG2 cells was decreased by both DON and DOM-1, but a significant difference among levels of each substance that affected albumin secretion was observed (228 μΜ for DOM-1 versus 0.9 μΜ for DON). In comparison, higher levels of DON were needed in our study to affect the motility characteristics of boar semen. Possibly, greater durability of boar semen in comparison with the aforementioned cell lines could be a part of the explanation for such a difference. Our study results are in agreement with findings that DOM-1 has substantially reduced toxicity and activity compared to DON, as seen for oxygen consumption, barrier function and MAPK induction in human intestinal epithelial cells at a concentration of 10 mM [13]. However, in contrast to studies with mouse 3T3 fibroblast, porcine intestinal epithelial cell lines and in porcine PBMCs in which DOM-1 did not affect cell viability [11,12], a study with bovine theca cells showed that DOM-1 (approximately 0.0036 μΜ, 4 days treatment) can induce apoptosis, probably through endoplasmic reticulum stress, whereas DON had no effect on the proportion of apoptotic cells at the same dosage level [16]. Variability among observations probably lies in differences in animal species, cell type, treatment duration and cultivation conditions used in different studies.

The present evaluation of ZEN and HZEN on boar semen demonstrated a negative effect of ZEN in the vast majority of kinetic parameters. Exposure to HZEN at an equimolar concentration failed to induce detrimental effects on boar semen characteristics. Therefore, similarly to DOM-1, HZEN cannot be categorized as a significant deteriorating factor of boar fertility.

HZEN is formed by enzymatic hydrolysis of the ester bond of ZEN’s lactone ring, which can further convert to decarboxylated HZEN (DHZEN) [38]. According to Fruhauf et al. [34], HZEN did not elicit an estrogenic response in an MCF-7 cell proliferation assay (0.01–500 nM) or an estrogen-sensitive yeast bioassay (1–10,000 nM). Additionally, HZEN did not increase vulva size or uterus weight in vivo (dietary exposure of prepubertal gilts to 4.58 mg ZEN/kg feed, as well as 4.84 HZEN mg/kg feed, for 4 weeks). RNA transcripts altered upon ZEN treatment (EBAG9, miR-135a-5p, miR-187-3p and miR-204-5p) remained unaffected by HZEN. Our findings in boar semen are in agreement with the above-mentioned observations; thus, the enzymatic conversion of ZEN to HZEN can be reported as a detoxification reaction.

Semen motility parameters are related to the fertilizing capacity of pig ejaculates [39]. Even though dosage levels used in the present study correspond to extremely high feed contamination levels, the possible chronic ingestion of lower doses by boars under field conditions should be considered when interpreting infertility cases in breeding stock. Mycotoxins have been shown to interfere at various levels and disrupt the activity of P450scc, 3βHSDs, 5α-reductases and/or P450aro in both males and females; thus, they can possibly affect the multienzymatic and hormonal aspects of spermatogenesis [40]. Additionally, possible mycotoxin-induced epigenetic modifications could also aggravate their effects on the male reproductive system, such as the hypothetical model of ZEN-induced mitochondrial damage on mouse Leydig tumor cells (MLTC-1). In that case, ZEN was capable of inducing increased energy production, excessive oxidative stress and inhibition of steroidogenesis and esterification, resulting in reduced hormone secretion [41]. Future studies with repeated or/and long-term mycotoxins exposure on male reproductive cells should provide additional respective answers.

## 4. Conclusions

Results of the present study confirm the toxic potency of DON and ZEN on boar semen kinetics, morphology and viability. Moreover, when tested in equimolar concentrations, the modified mycotoxins DOM-1 and HZEN do not elicit toxic effects on boar semen in vitro. Our study provides further evidence that conversion of DON to DOM-1, as well as of ZEN to HZEN, represents a detoxification process, expanding previous findings in other organ systems to the male reproductive system.

## 5. Materials and Methods

This study was approved by the Research Committee of the Aristotle University of Thessaloniki, Greece (Code No.: 92520, Scientific Responsible: P.D. Tassis). The tests were carried out in the Unit of Biotechnology of Reproduction of the Farm Animals Clinic, School of Veterinary Medicine, Aristotle University of Thessaloniki, Greece.

### 5.1. Samples Origin and Procedures

In this study, we followed testing procedures similar to a previous study by our group, with particular details on handling, sample preparation, origin of boars and analysis of boar feed available, in the respective publication [7]. Semen samples from active boars were used (Duroc × Pietrain hybrid, 13–14 months old at the start of the study). Results of feed analysis performed by LC–MS/MS for aflatoxin B1, DON, ZEN, ochratoxin A, T2 toxin [42] and fumonisins [43] showed a lack or traces of those mycotoxins [7]. At each sampling (use of the gloved-hand method for semen collection), the final sample was prepared after pooling two different boar ejaculations. At the pig farm, a commercially available extender (OPTIM-I.A^®^, Magapol, Spain) was used to perform one-step semen dilution to a final concentration of 30 × 10^6^ sperm/mL. Samples (25 in total) that were included in the tests met the following quality criteria: viability > 75%, total motility > 60%, concentration > 100 × 10^6^ sperm/mL, morphological abnormalities < 15%.

The standards of the parent mycotoxins (DON and ZEN) were purchased from Romer Labs (Tulln, Austria; >99% purity). Respective metabolites (DOM-1 and HZEN, respectively) were produced according to procedures described by Schwarz-Zimmerman et al. [44,45] and Fruhauf et al. [34]. All mycotoxins were stored at −18 °C until use. Stock solutions were prepared with dissolvement of respective mycotoxins quantities in DMSO (D-4540, Sigma-Aldrich Corporation, Saint Louis, MO, USA; >99.5% purity), reaching final concentrations of 7.23 µM DON, 8.97 µM ZEN, 7.23 µM DOM-1 or 8.97 µM HZEN. Appropriate amounts of stock solutions were added to semen samples, (1 mL aliquots at the pretrial and 3 mL at the main trial), followed by incubation in sterilized 10 mL tubes for 4 h (38.5 °C, 5% CO_2_, 100% humidity). At the time points of 0, 1, 2, 3 and 4 h of incubation, each analysis test was performed at an appropriate semen volume. As regards initial effects presented on time point 0 h, those refer to practical conditions of a time period of seconds from the addition of the respective mycotoxin concentration to the semen sample until its evaluation, thus representing an acute effect.

### 5.2. Trial Procedures

A preliminary evaluation regarding DMSO, DON and ZEN has been presented in a previous publication by our group [7]. Briefly, results of that part of the study (CASA analysis of five replicates) demonstrated that the MiD levels that could significantly reduce semen progressive motility after 1 h of incubation were 50.6 μM DON and 62.8 μM ZEN, whereas the DMSO level of 0.7% did not affect the above-mentioned parameter in comparison with control semen (without DMSO addition). Furthermore, an additional preliminary evaluation of the effects of 50.6 μM DOM-1 and 62.8 μM HZEN on progressive motility (five replicates) after 1 h of incubation, in comparison with DMSO-treated semen, following similar CASA procedures as for the main toxins, was carried out. Results of metabolites’ pretrial evaluation showed the absence of significant effects on progressive motility of spermatozoa.

The main trial was carried out in ten replicates for each evaluation, according to appropriate sample size calculation, and included two evaluations, one for each pair of related mycotoxins (i.e., DON-DOM-1 in the first and ZEN-HZEN in the second evaluation). Effects of DON, ZEN and their respective metabolites were evaluated at MiD levels with regard to kinetic parameters (CASA), morphology, viability, membrane biochemical function, and chromatin integrity of semen. Test groups in each study part were:

(i) Evaluation of DON and DOM-1 on boar semen characteristics: (a) Control group (semen without addition of DMSO or mycotoxins); (b) DMSO group (0.7% *v*/*v* DMSO); (c) DON group (addition of 50.6 μM DON); (d) DOM-1 group (addition of 50.6 μM DOM1).
(ii) Evaluation of ZEN and HZEN on boar semen characteristics: (a) Control group [similar to the above-mentioned evaluation (i)]; (b) DMSO group [similar to the above-mentioned evaluation (i)]; (c) ZEN group (addition of 62.8 μM ZEN); (d) HZEN group (addition of 62.8 μM HZEN).

The following methods were performed for the evaluation of semen characteristics, as previously described [7]:

(a) Motility/kinetics parameters of sperm (total motility, progressive motility, immotile, rapid, medium, slow spermatozoa, curvilinear velocity (VCL), straight-line velocity (VSL), average path velocity (VAP), lateral head displacement (ALH), beat/cross frequency (BCF), hyperactivation, straightness (STR), linearity (LIN), wobble (WOB)) with the use of CASA (Sperm Class Analyser^®^ v.5.2.0.0., Microptic S.L., Automatic Diagnostic Systems, Barcelona, Spain) and a microscope (X100; AXIO Scope A1, Zeiss, Jena, Germany) accomplished with a heating stage.
(b) The SpermBlue staining method (SpermBlue^®^ 08029, Microptic S.L., Barcelona, Spain) was used for the evaluation of sperm morphology, according to the manufacturer’s instructions.
(c) The double fluorescent stain calcein-AM (C-AM; 1 mmol/L) and propidium iodide (PI; 0.75 mmol/L) was appropriately utilized for sperm viability assessment.
(d) The hypoosmotic swelling test (HOST) was performed as previously demonstrated [46] under slight modification for the assessment of sperm membrane functional status.
(e) The AOT was used to evaluate sperm nuclear chromatin integrity. AOT measures the susceptibility of sperm nuclear DNA to acid-induced denaturation in situ through quantification of the metachromatic shift of acridine orange fluorescence from green (native DNA) to red (denatured DNA).

### 5.3. Statistical Analysis

Statistical analysis followed the methodology described in a previous study from our group [7]. Sample size (N) determination for the main trial was performed with an a priori power analysis (statistical software G*Power, version 3.1.9) [47,48]. In that case, sample size was computed as a function of the required power level (1-β), the prespecified significance level (α) and the population effect size to be detected with probability (1-β). The power and significance levels were set at β = 0.80 and a = 0.05 [47].

The examination of the effects for the two factors of interest, which were (i) mycotoxin effect and (ii) time, on the parameters under investigation for both the pre- and the main trials of the study was conducted via the linear mixed-effects (LME) models [49]. Regarding the insertion of the main and interaction effects affecting the response (i.e., examined parameters), we followed the structured guidelines proposed by Zuur et al. [50], whereas for the statistical analysis of the collected data, we utilized the statistical programming language R [51]. More specifically, the fitting of all models was based on the function lmer that can be found in the lme4 library, whereas for the identification of the factors presenting a significant effect on the response, we used the backward elimination functionality provided in the lmerTest library [52]. The inferential process was based on the F-test and *p*-values computed by the Kenward–Roger approximation [53] and the alpha level for all statistical hypothesis testing procedures was set at 0.05. Due to the reduced number of positive cases, sperm nuclear chromatin integrity results did not undergo statistical evaluation.

## Figures and Tables

**Table 1:** Descriptive statistics of major kinetic CASA measurements of boar semen after DON or DOM-1 exposure (mean values ± standard deviation) at each observation time point (0–4 h). Number of replicates = 10 in each test.

Treatments ^#^	0 h	1 h	2 h	3 h	4 h
**Immotile spermatozoa (%)** *					
DMSO	5.42 ± 4.19	5.06 ± 2.06	7.87 ± 3.65	9.83 ± 6.82	10.76 ± 6.34
DON	6.65 ± 3.76	7.22 ± 5.22	11.54 ± 3.71	12.40 ± 6.10	14.01 ± 6.74
DOM-1	7.11 ± 6.03	6.18 ± 3.52	10.30 ± 3.36	11.51 ± 8.02	11.99 ± 4.81
DON effect on immotile spermatozoa without treatment X time interaction: *p* = 0.004 DON vs. DMSO					
**Nonprogressive motile spermatozoa (%)** **^ǁ^**					
DMSO	25.37 ± 6.75	19.65 ± 4.30	18.79 ± 3.58	17.72 ± 4.56	17.35 ± 3.19
DON	25.09 ± 7.47	19.84 ± 3.48	19.35 ± 4.71	19.26 ± 4.65	19.58 ± 5.11
DOM-1	24.31 ± 5.11	20.34 ± 2.76	19.88 ± 4.90	17.39 ± 3.76	17.79 ± 4.20
**Progressive motile spermatozoa (%)** *					
DMSO	69.21 ± 10.04	75.29 ± 5.23	73.34 ± 5.36	72.45 ± 8.78	71.89 ± 7.43
DON	68.26 ± 10.24	72.94 ± 6.36	69.11 ± 5.24	68.34 ± 8.58	66.41 ± 6.31
DOM-1	68.59 ± 9.57	73.47 ± 5.22	69.82 ± 5.15	71.10 ± 7.72	70.22 ± 6.45
DON effect on progressive motile spermatozoa without treatment X time interaction: *p* = 0.016 DON vs. DMSO					
**Rapid (%) ^ǁ^**					
DMSO	69.23 ± 11.08	53.31 ± 10.19	51.05 ± 11.41	46.15 ± 13.15	43.88 ± 15.38
DON	67.63 ± 9.83	50.89 ± 14.57	42.74 ± 10.44	42.22 ± 12.42	39.90 ± 8.33
DOM-1	66.17± 12.78	53.01 ± 12.85	44.90 ± 10.55	43.35 ± 10.78	44.51 ± 12.20
**Medium (%)** **^ǁ^**					
DMSO	16.02 ± 10.04	27.45 ± 7.73	27.95 ± 6.53	30.98 ± 9.03	32.96 ± 11.24
DON	16.64 ± 9.64	27.97 ± 10.34	30.56 ± 9.4	30.46 ± 8.71	32.24 ± 6.98
DOM-1	16.62 ± 8.47	27.09 ± 8.35	29.22 ± 7.04	31.94 ± 9.33	30.72 ± 8.32
**Slow (%)** **^ǁ^**					
DMSO	9.32 ± 3.53	14.19 ± 4.10	13.13 ± 4.92	13.03 ± 5.28	12.40 ± 3.15
DON	9.09 ± 3.51	13.93 ± 5.15	15.17 ± 4.21	14.92 ± 5.01	13.85 ± 2.88
DOM-1	10.10 ± 4.59	13.72 ± 4.24	15.58 ± 4.55	13.20 ± 3.98	12.79 ± 3.25

**^#^** Treatments: DMSO = 0.7% (*v*/*v*); DON = 50.6 μΜ; DOM-1 = 50.6 μΜ. * Differences among DON and DMSO of immotile and progressive motile spermatozoa mean values are reported without superscripts, since they refer to significant main effect (*p* < 0.05) of treatment (mycotoxin) on the response variable, without significant interaction term Treatment × Time; thus, differences refer to the total observation period. **^ǁ^** *p* > 0.05 for all comparisons among groups at each time point and respective parameter.

**Table 2:** Descriptive statistics of velocity and trajectory CASA measurements of boar semen after DON or DOM-1 exposure (mean values ± standard deviation) at each observation time point (0–4 h). Number of replicates = 10 in each test.

Treatments ^#^	0 h	1 h	2 h	3 h	4 h
**VCL (Curvilinear velocity; μm/s)** **^ǁ^**					
DMSO	74.31 ± 17.82	51.14 ± 6.41	50.50 ± 6.00	47.75 ± 6.53	47.61 ± 7.03
DON	72.91 ± 16.84	51.88 ± 8.65	46.97 ± 6.56	46.36 ± 8.70	45.70 ± 4.68
DOM-1	70.89 ± 13.93	52.46 ± 9.14	46.67 ± 5.35	46.59 ± 6.22	47.66 ± 6.88
**VSL (Straight-line velocity; μm/s)** **^ǁ^**					
DMSO	32.02 ± 2.01	35.32 ± 3.63	36.69 ± 3.53	36.13 ± 5.42	36.37 ± 5.88
DON	31.44 ± 2.23	35.53 ± 3.24	35.46 ± 5.38	35.86 ± 7.64	34.75 ± 4.12
DOM-1	31.45 ± 2.82	34.71 ± 3.30	34.91 ± 4.72	35.81 ± 5.51	36.55 ± 5.84
**VAP (Average path velocity; μm/s)** **^ǁ^**					
DMSO	49.74 ± 6.48	42.92 ± 4.20	44.08 ± 4.40	42.66 ± 5.98	42.76 ± 6.35
DON	48.96 ± 6.36	43.37 ± 4.81	41.55 ± 5.76	41.72 ± 8.17	40.77 ± 4.25
DOM-1	47.21 ± 6.16	43.03 ± 4.85	40.98 ± 4.87	41.80 ± 5.83	42.77 ± 6.18
**LIN (Linearity; %)** **^ǁ^**					
DMSO	45.78 ± 12.67	69.29 ± 3.74	72.90 ± 4.07	75.60 ± 3.09	76.31 ± 2.88
DON	45.68 ± 12.56	69.29 ± 6.14	75.47 ± 3.24	77.20 ± 3.49	76.05 ± 3.91
DOM-1	45.74 ± 8.78	67.15 ± 7.93	74.76 ± 3.85	76.77 ± 3.76	76.63 ± 3.52
**STR (Straightness; %)** **^ǁ^**					
DMSO	65.49 ± 10.19	82.25 ± 1.13	83.30 ± 2.68	84.64 ± 2.65	84.92 ± 2.01
DON	65.33 ± 10.45	82.11 ± 3.19	85.23 ± 1.44	85.81 ± 2.30	85.19 ± 2.75
DOM-1	67.31 ± 7.74	80.87 ± 4.34	85.08 ± 2.42	85.55 ± 2.31	85.32 ± 2.60
**Wobble (WOB; %)** **^ǁ^**					
DMSO	68.83 ± 9.41	84.22 ± 3.94	87.46 ± 2.56	89.30 ± 1.25	89.83 ± 1.42
DON	68.84 ± 8.61	84.25 ± 4.72	88.52 ± 2.73	89.93 ± 1.87	89.22 ± 2.01
DOM-1	67.63 ± 7.37	82.78 ± 5.66	87.85 ± 3.40	89.71 ± 2.65	89.78 ± 1.95
**ALH (Amplitude of lateral head displacement; μm)** **^ǁ^**					
DMSO	2.15 ± 0.48	1.51 ± 0.12	1.44 ± 0.14	1.34 ± 0.09	1.32 ± 0.09
DON	2.04 ± 0.39	1.52 ± 0.19	1.35 ± 0.08	1.29 ± 0.13	1.30 ± 0.07
DOM-1	1.98 ± 0.33	1.56 ± 0.21	1.35 ± 0.06	1.31 ± 0.09	1.31 ± 0.10
**BCF (Beat/Cross Frequency; Hz)** **^ǁ^**					
DMSO	12.53 ± 2.51	10.02 ± 1.33	9.24 ± 0.74	8.85 ± 0.54	8.89 ± 0.62
DON	12.88 ± 2.39	9.88 ± 1.42	9.00 ± 0.72	8.59 ± 0.73	8.94 ± 0.25
DOM-1	13.04 ± 2.55	9.99 ± 1.55	9.38 ± 1.14	9.06 ± 0.67	9.05 ± 0.64
**Hyperactive (%)** **^ǁ^**					
DMSO	2.33 ± 0.88	0.88 ± 0.63	0.70 ± 0.57	0.46 ± 0.53	0.18 ± 0.13
DON	1.93 ± 0.72	0.97 ± 1.07	0.27 ± 0.20	0.19 ± 0.25	0.19 ± 0.21
DOM-1	2.05 ± 1.09	1.07 ± 0.81	0.39 ± 0.39	0.25 ± 0.26	0.36 ± 0.44

**^#^** Treatments: DMSO = 0.7% (*v*/*v*); DON = 50.6 μΜ; DOM-1 = 50.6 μΜ. **^ǁ^** *p* > 0.05 for all comparisons among groups at each time point and respective parameter.

**Table 3:** Descriptive statistics of boar semen traits after DON and DOM-1 exposure (mean values ± standard deviation) at each observation time (0 h–4 h of incubation). Number of replicates = 10 in each test.

Morphology (% Spermatozoa without Abnormalities) ^ǁ^					
Treatments ^#^	0 h	1 h	2 h	3 h	4 h
DMSO	94.95 ± 2.76	93.65 ± 4.90	89.95 ± 5.28	86.20 ± 7.68	84.25 ± 7.87
DON	95.35 ± 3.15	92.90 ± 4.55	86.75 ± 6.80	82.60 ± 10.20	82.75 ± 9.14
DOM-1	95.50 ± 2.35	92.70 ± 6.19	88.90 ± 5.88	87.05 ± 7.83	84.20 ± 7.80
**Morphology (% spermatozoa with head abnormalities)** **^ǁ^**					
DMSO	3.65 ± 2.56	4.85 ± 4.22	8.40 ± 4.80	12.30 ± 7.57	14.65 ± 7.69
DON	2.85 ± 3.03	5.70 ± 4.52	11.35 ± 6.75	16.35 ± 10.27	16.15 ± 9.13
DOM-1	3.40 ± 2.57	6.10 ± 6.02	9.70 ± 5.35	12.15 ± 7.76	14.65 ± 7.88
**Viability (% live spermatozoa)** **^ǁ^**					
DMSO	90.15 ± 2.81	85.85 ± 4.47	84.45 ± 3.50	83.90 ± 3.45	84.60 ± 3.71
DON	90.65 ± 3.10	84.00 ± 5.73	84.50 ± 5.32	82.25 ± 5.34	81.10 ± 5.40
DOM-1	90.20 ± 1.92	85.85 ± 3.69	85.30 ± 5.39	85.30 ± 3.60	85.50 ± 4.87
**Hypoosmotic Swelling Test (HOST, % spermatozoa with swollen tails)** **^ǁ^**					
	**0 h**		**1 h**		**4 h**
DMSO	17.70 ± 5.29		11.40 ± 5.63		8.10 ± 4.08
DON	18.65 ± 6.42		10.50 ± 5.46		7.65 ± 5.30
DOM-1	16.75 ± 5.60		10.75 ± 5.86		7.65 ± 4.42

^#^ Treatments: DMSO = 0.7% (*v*/*v*); DON = 50.6 μΜ; DOM-1 = 50.6 μΜ. ^ǁ^ *p* > 0.05 for all comparisons among groups.

**Table 4:** Descriptive statistics of major kinetic CASA measurements of boar semen after ZEN or HZEN exposure (mean values ± standard deviation) at each observation time point (0–4 h). Number of replicates = 10 in each test.

Treatments **^#^**	0 h	1 h	2 h	3 h	4 h	*p* * (DMSO vs. Mycotoxin Treatment)
**Immotile spermatozoa (%)**						
DMSO	7.15 ± 5.82 ^ab^	6.66 ± 4.03 ^b^	7.98 ± 3.56 ^b^	13.87 ± 5.87 ^b^	15.07 ± 4.86 ^b^	
ZEN	19.02 ± 11.33 ^a^	31.12 ± 16.72 ^a^	37.19 ± 16.44 ^a^	37.11 ± 14.21 ^a^	39.09 ± 11.75 ^a^	1–4 h: <0.001 **
HZEN	4.64 ± 3.06 ^b^	8.06 ± 4.76 ^b^	12.65 ± 7.86 ^b^	14.95 ± 6.23 ^b^	16.90 ± 5.60 ^b^	
**Nonprogressive motile spermatozoa (%)**						
DMSO	25.47 ± 8.00	19.00 ± 3.58	20.57 ± 5.53	20.41 ± 9.46	19.75 ± 4.32	
ZEN	32.69 ± 4.22	29.75 ± 7.96	30.40 ± 7.79	33.95 ± 8.22	36.49 ± 10.27	<0.001
HZEN	24.75 ± 7.71	19.93 ± 4.22	20.97 ± 4.33	23.82 ± 7.78	20.27 ± 5.46	
**Progressive motile spermatozoa (%)**						
DMSO	67.38 ± 12.34 ^b^	74.33 ± 5.62 ^b^	71.45 ± 6.26 ^b^	65.72 ± 12.81 ^b^	65.19 ± 6.05 ^b^	
ZEN	48.29 ± 13.72 ^a^	39.13 ± 22.23 ^a^	32.40 ± 21.80 ^a^	28.93 ± 17.76 ^a^	24.42 ± 14.10 ^a^	0 h = 0.024; 1–4 h: <0.001 **
HZEN	70.62 ± 9.34 ^b^	72.01 ± 5.69 ^b^	66.38 ± 8.93 ^b^	61.24 ± 12.34 ^b^	62.83 ± 7.78 ^b^	
**Rapid (%)**						
DMSO	65.44 ± 9.94	54.44 ± 10.59	50.13 ± 13.73	42.53 ± 17.10	38.54 ± 10.21	
ZEN	42.40 ± 13.74	24.65 ± 16.92	20.18 ± 18.12	13.53 ± 12.12	10.36± 8.27	<0.001
HZEN	72.16 ± 6.10	52.36 ± 10.42	41.21 ± 11.36	35.19 ± 15.85	35.25 ± 9.42	
**Medium (%)**						
DMSO	17.59 ± 9.60 ^a^	25.51 ± 7.87 ^a^	26.68 ± 10.41 ^b^	26.87 ± 7.76 ^ab^	30.13 ± 9.42 ^a^	
ZEN	17.89 ± 4.83 ^a^	18.46 ± 8.70 ^a^	15.69 ± 5.98 ^a^	18.80 ± 8.70 ^b^	16.76 ± 7.66 ^b^	2 h = 0.019, 4 h = 0.001 **
HZEN	14.93 ± 6.32 ^a^	25.49 ± 8.05 ^a^	29.92 ± 10.24 ^b^	31.03 ± 10.41 ^a^	31.31 ± 9.64 ^a^	
**Slow (%)**						
DMSO	9.81 ± 3.34	13.39 ± 3.64	15.21 ± 6.10	16.73 ± 10.51	16.27 ± 4.48	
ZEN	20.69 ± 6.12	25.76 ± 9.34	26.93 ± 9.01	30.56 ± 8.49	33.79 ± 10.06	<0.001
HZEN	8.28 ± 3.22	14.10 ± 4.65	16.23 ± 4.30	18.84 ± 7.90	16.54 ± 5.61	

**^#^** Treatments: DMSO = 0.7% (*v*/*v*); ZEN = 62.8 μΜ; HZEN = 62.8 μΜ. ^a,b^ Mean values with different superscripts in the same column differ significantly (*p* < 0.05). * When *p*-values are reported without superscripts in relevant parameter mean values: significant main effect (*p* < 0.05) of treatment (mycotoxin) on the response variable, without significant interaction term Treatment × Time; thus, differences refer to the total observation period. ** When *p*-values are reported and superscripts (a, b) are placed in relevant parameter mean values: significant main effect (*p* < 0.05) of treatment (mycotoxin) on the response variable, with a significant interaction term Treatment × Time on the response variable present; thus, differences refer to specific time points [0–4 hours (0–4 h) of investigation]. *p*-values ZEN vs. HZEN: Immotile 0 h = 0.007, 1–4 h: <0.001; Nonprogressive motile: <0.001; Progressive: 0 h = 0.002, 1–4 h: <0.001; Medium: 2 h, 4 h = <0.001, 3 h = 0.004; Rapid; Slow: <0.001.

**Table 5:** Descriptive statistics of velocity and trajectory CASA measurements of boar semen after ZEN or HZEN exposure (mean values ± standard deviation) at each observation time point (0–4 h). Number of replicates = 10 in each test.

Treatments ^#^	0 h	1 h	2 h	3 h	4 h	*p* * (DMSO vs. Mycotoxin Treatment)
**VCL (Curvilinear velocity; μm/s)**						
DMSO	70.90 ± 15.23	52.74 ± 7.18	51.39 ± 9.14	46.33 ± 9.81	44.94 ± 5.93	
ZEN	52.55 ± 8.96	36.49 ± 11.99	33.35 ± 13.25	29.68 ± 9.24	27.72 ± 6.46	<0.001
HZEN	73.30 ± 12.20	52.15 ± 6.70	46.85 ± 7.84	43.62 ± 8.35	44.07 ± 6.36	
**VSL (Straight-line velocity; μm/s)**						
DMSO	29.84 ± 2.95 ^a^	35.79 ± 4.89 ^a^	35.75 ± 5.00 ^a^	34.93 ± 7.67 ^a^	33.82 ± 4.17 ^a^	
ZEN	20.83 ± 4.91 ^a^	20.34 ± 9.83 ^b^	18.45 ± 9.41 ^b^	16.77 ± 8.18 ^b^	15.43 ± 7.10 ^b^	1–4 h: <0.001 **
HZEN	32.28 ± 3.63 ^a^	34.10 ± 3.00 ^a^	32.84 ± 6.22 ^a^	31.20 ± 6.26 ^a^	32.43 ± 5.57 ^a^	
**VAP (Average path velocity; μm/s)**						
DMSO	46.38 ± 4.59	43.63 ± 5.56	43.08 ± 6.07	40.81 ± 8.46	39.62 ± 4.60	
ZEN	32.47 ± 6.48	26.42 ± 11.48	24.00 ± 11.38	21.76 ± 9.12	20.13 ± 7.51	<0.001
HZEN	49.01 ± 3.59	42.14 ± 3.21	39.48 ± 6.67	37.26 ± 7.09	38.22 ± 5.96	
**LIN (Linearity; %)**						
DMSO	46.38 ± 4.59 ^a^	43.63 ± 5.56 ^a^	43.08 ± 6.07 ^a^	40.81 ± 8.46 ^a^	39.62 ± 4.60 ^a^	
ZEN	32.47 ± 6.48 ^a^	26.42 ± 11.48 ^b^	24.00 ± 11.38 ^b^	21.76 ± 9.12 ^b^	20.13 ± 7.51 ^b^	1–4 h: <0.001 **
HZEN	49.01 ± 3.59 ^a^	42.14 ± 3.21 ^a^	39.48 ± 6.67 ^a^	37.26 ± 7.09 ^a^	38.22 ± 5.96 ^a^	
**STR (Straightness; %)**						
DMSO	64.99 ± 9.52	81.94 ± 2.46	83.04 ± 2.35	85.39 ± 2.66	85.32 ± 1.61	
ZEN	63.90 ± 5.39	75.10 ± 5.34	76.02 ± 3.07	74.98 ± 6.42	74.86 ± 6.03	<0.001
HZEN	66.03 ± 7.41	80.91 ± 2.87	82.98 ± 2.91	83.63 ± 2.30	84.68 ± 2.78	
**Wobble (WOB; %)**						
DMSO	67.01 ± 8.60 ^a^	82.89 ± 3.88 ^a^	84.35 ± 4.19 ^a^	88.16 ± 2.16 ^a^	88.31 ± 2.12 ^a^	
ZEN	68.84 ± 8.61 ^a^	84.25 ± 4.72 ^b^	88.52 ± 2.73 ^b^	89.93 ± 1.87 ^b^	89.22 ± 2.01 ^b^	1–4 h = <0.001 **
HZEN	68.04 ± 8.40 ^a^	81.42 ± 6.11 ^a^	84.44 ± 5.92 ^a^	85.47 ± 4.04 ^a^	86.72 ± 5.16 ^a^	
**ALH (Amplitude of lateral head displacement; μm)** **^ǁ^**						
DMSO	2.08 ± 0.49	1.53 ± 0.13	1.49 ± 0.18	1.35 ± 0.11	1.32 ± 0.10	
ZEN	2.07 ± 0.31	1.60 ± 0.11	1.46 ± 0.26	1.41 ± 0.18	1.42 ± 0.14	
HZEN	2.18 ± 0.46	1.56 ± 0.13	1.43 ± 0.12	1.35 ± 0.13	1.33 ± 0.14	
**BCF (Beat/Cross Frequency; Hz)**						
DMSO	12.57 ± 1.76	10.48 ± 1.36	10.11 ± 1.60	9.31 ± 1.00	9.26 ± 0.93	
ZEN	10.25 ± 1.19	8.11 ± 2.02	7.76 ± 1.61	7.52 ± 1.14	7.88 ± 0.76	<0.001
HZEN	12.52 ± 1.56	10.45 ± 1.83	9.51 ± 1.33	9.19 ± 1.12	9.21 ± 0.80	
**Hyperactive (%)**						
DMSO	1.78 ± 0.82	0.95 ± 0.82	1.03 ± 0.79	0.42 ± 0.51	0.31 ± 0.32	
ZEN	1.16 ± 0.76	0.62 ± 0.57	0.79 ± 1.59	0.36 ± 0.49	0.12 ± 0.19	0.022
HZEN	2.63 ± 1.00	0.96 ± 0.64	0.72 ± 0.60	0.49 ± 0.53	0.49 ± 0.62	

**^#^** Treatments: DMSO = 0.7% (*v*/*v*); ZEN = 62.8 μΜ; HZEN = 62.8 μΜ. ^a,b^ Mean values with different superscripts in the same column differ significantly (*p* < 0.05). * When *p*-values are reported without superscripts in relevant parameter mean values: significant main effect (*p* < 0.05) of treatment (mycotoxin) on the response variable, without significant interaction term Treatment × Time; thus, differences refer to the total observation period. ** When *p*-values are reported and superscripts (a, b) are placed in relevant parameter mean values: significant main effect (*p* < 0.05) of treatment (mycotoxin) on the response variable, with a significant interaction term Treatment × Time on the response variable present; thus, differences refer to specific time points [0–4 hours (0–4 h) of investigation]. *p*-values ZEN vs. HZEN:; VCL; VAP; STR: <0.001; VSL: 0 h = 0.005; 1–4 h: <0.001; LIN: 1 h = 0.002, 2–4 h: <0.001; WOB: 1–4 h: <0.001; Hyperactive: 0.005. ^ǁ^ *p* > 0.05 for all comparisons among groups.

**Table 6:** Descriptive statistics of boar semen traits after ZEN and HZEN exposure (mean values ± standard deviation) at each observation time (0 h–4 h of incubation). Number of replicates = 10 in each test.

Morphology (% Spermatozoa without Abnormalities)						
Treatments ^**#**^	0 h	1 h	2 h	3 h	4 h	*p* * (DMSO vs. Mycotoxin Treatment)
DMSO	87.70 ± 8.10	83.60 ± 11.90	79.60 ± 13.30	75.60 ± 11.82	75.20 ± 14.57	
ZEN	77.40 ± 18.19	71.60 ± 21.58	69.00 ± 20.52	68.00 ± 21.29	63.10 ± 21.15	0.002
HZEN	87.10 ± 5.90	81.60 ± 13.13	77.10 ± 16.05	69.40 ± 15.94	68.30 ± 16.26	
**Morphology (% spermatozoa with head abnormalities)**						
DMSO	9.20 ± 8.32	15.50 ± 11.75	17.80 ± 14.27	21.80 ± 13.74	23.40 ± 15.25	
ZEN	20.60 ± 18.95	27.10 ± 22.11	28.70 ± 21.64	29.80 ± 22.57	34.60 ± 21.91	0.046
HZEN	11.40 ± 6.87	16.60 ± 14.07	21.70 ± 15.60	30.40 ± 17.24	31.10 ± 16.72	
**Viability (% live spermatozoa)**						
DMSO	85.50 ± 7.17 ^a^	79.90 ± 12.18 ^a^	76.70 ± 13.57 ^a^	72.60 ± 11.12 ^a^	71.50 ± 12.72 ^a^	
ZEN	65.10 ± 17.44 ^b^	49.10 ± 19.36 ^b^	39.60 ± 17.73 ^b^	36.20 ± 14.83 ^b^	32.30 ± 13.54 ^b^	0–4 h: <0.001 **
HZEN	85.40 ± 7.09 ^a^	78.90 ± 9.97 ^a^	75.10 ± 11.55 ^a^	67.00 ± 15.78 ^a^	58.90 ± 18.05 ^a^	
**Hypoosmotic Swelling Test (HOST, % spermatozoa with swollen/coiled tails)** ^ǁ^						
	**0 h**		**1 h**		**4 h**	
DMSO	20.70 ± 8.81		10.70 ± 4.95		9.50 ± 4.67	
ZEN	15.60 ± 6.70		11.00 ± 4.35		7.00 ± 4.29	
HZEN	16.20 ± 7.18		10.20 ± 5.22		7.20 ± 3.61	

^**#**^ Treatments: DMSO = 0.7% (*v*/*v*); ZEN = 62.8 μΜ; HZEN = 62.8 μΜ. ^a,b^ Mean values with different superscripts in the same column differ significantly (*p* < 0.05). * When *p*-values are reported without superscripts in relevant parameter mean values: significant main effect of treatment (mycotoxin) on the response variable, without significant interaction term Treatment × Time; thus, differences refer to the total observation period. ** When *p*-values are reported and superscripts (a, b) are placed in relevant parameter mean values: significant main effect (*p* < 0.05) of treatment (mycotoxin) on the response variable, with a significant interaction term Treatment × Time on the response variable present; thus, differences refer to specific time points [0–4 hours (0–4 h) of investigation]. *p*-values ZEN vs. HZEN: Spermatozoa without abnormalities: 0.084; Viability: 0–4 h: <0.001. ^ǁ^ *p* > 0.05 for all comparisons among groups.

## Data Availability

The data presented in this study are available on reasonable request from the corresponding author after permission of the Funding Body.
